# Supplementary material for: Genetic tracing of the illegal trade of the white-bellied pangolin (Phataginus tricuspis) in western Central Africa
Source: Sci Rep. 2024 Jun 7;14:13131. doi: 10.1038/s41598-024-63666-9 (PMC11161582; doi:10.1038/s41598-024-63666-9)
Supplement: Supplementary file 1 — Supplementary Information 1. [file 41598_2024_63666_MOESM1_ESM.docx]

Supplementary Table S1. Samples of white-bellied pangolins used in this study, including information on their geographic referencing, lineage / cluster assignment, tracing and genotypes [Excel File].

In red, individuals identified as cyto-nuclear hybrids or admixed individuals between the two lineages present in western central Africa.

Lineages as defined in Gaubert et al. (2016): WCA = Western Central Africa; Gab = Gabon.

Populations: GES = southern Equatorial Guinea; Mt Cam = National Park of Mt Cameroon; PNCM = National Park of Campo Ma’an

Supplementary Table S2. Genetic diversity estimates among white-bellied pangolin lineages based on cytochrome *b*.

|  | **WCA** | ***WCA (Gaubert et al., 2016)*** | **Gabon** | **Dahomey Gap** | **Western Africa** | **Central Africa** | **Ghana** |
| --- | --- | --- | --- | --- | --- | --- | --- |
| Number of sequences | 552 | *152* | 13 | 14 | 12 | 14 | 4 |
| Number of sites | 381 | *402* | 381 | 402 | 402 | 402 | 402 |
| Number of Haplotypes, *h* | 67 | *31* | 8 | 5 | 6 | 7 | 3 |
| Haplotype diversity, *Hd* | 0.830 | *0.811* | 0.897 | 0.703 | 0.682 | 0.879 | 0.833 |
| Nucleotide diversity, π | 0.010 | *0.006* | 0.019 | 0.003 | 0.002 | 0.007 | 0.003 |

Supplementary Table S3. Locus significantly affected by null alleles under the assumption of Hardy-Weinberg equilibrium (in bold, after Bonferroni correction), in white-bellied pangolins from the Foumbot population (WCA; N = xx).

Adjusted allele’s frequencies of amplified allele’s bases on the four correction methods (Oosterhout, Chakraborty, Brookfield 1 and Brookfield 2) of null allele estimation.

| **Locus** | **Null Present** | **Oosterhout** | **Chakraborty** | **Brookfield 1** | **Brookfield 2** |
| --- | --- | --- | --- | --- | --- |
| PT1162028 | no | 0,0369 | 0,039 | 0,0273 | 0,0273 |
| PT1753627 | no | -0,0448 | -0,0382 | -0,0346 | 0 |
| **PT796077** | **yes** | 0,1578 | 0,1924 | 0,1454 | 0,1454 |
| **PT1973508** | **yes** | 0,083 | 0,0904 | 0,0769 | 0,0769 |
| **PT839522** | **yes** | 0,2901 | 0,4504 | 0,2469 | 0,2469 |
| PT464918 | no | -0,0262 | -0,0208 | -0,0181 | 0 |
| **PT1453906** | **yes** | 0,1384 | 0,1748 | 0,1153 | 0,1153 |
| PT34432 | no | -0,0473 | -0,0406 | -0,0338 | 0 |
| **PT1594892** | **yes** | 0,0894 | 0,0984 | 0,0803 | 0,0803 |
| PT308752 | no | -0,0151 | -0,0134 | -0,0121 | 0 |
| PT1669238 | no | 0,0407 | 0,0485 | 0,0391 | 0,0391 |
| PT1225378 | no | -0,0203 | -0,0255 | -0,0126 | 0 |
| PT739516 | no | 0,0434 | 0,0649 | 0,0315 | 0,0315 |
| PT619913 | no | 0,0905 | 0,1021 | 0,0654 | 0,0654 |
| **PT338821** | **yes** | 0,1182 | 0,1296 | 0,0962 | 0,0962 |
| **PT1849728** | **yes** | 0,1299 | 0,172 | 0,1011 | 0,1794 |
| PT378852 | no | 0,0966 | 0,1065 | 0,0607 | 0,1553 |
| **PT353755** | **yes** | 0,2079 | 0,2678 | 0,1799 | 0,2329 |
| PT276641 | no | -0,043 | -0,027 | -0,0209 | 0 |
| PT2019332 | no | -0,0058 | -0,0007 | -0,0007 | 0 |

Supplementary Table S4. Deviation of genotypic frequencies from those expected under Hardy-Weinberg equilibrium in white-bellied pangolins from the Foumbot population.

Significant values are in bold (after Bonferroni correction).

| **Locus** | PT1162028 | PT1753627 | PT796077 | PT1973508 | PT839522 | PT464918 | PT1453906 | PT34432 | PT1594892 | PT308752 |
| --- | --- | --- | --- | --- | --- | --- | --- | --- | --- | --- |
|  | 0.667 | 0.997 | 0.055 | 0.402 | **0.000***** | **0.000***** | 0.189 | 1.000 | **0.001*** | 0.604 |
|  | | | | | | | | | | |
| **Locus** | PT1669238 | PT1225378 | PT739516 | PT619913 | PT338821 | PT1849728 | PT378852 | PT353755 | PT276641 | PT2019332 |
|  | 0.091 | 0.908 | 0.015 | 0.063 | 0.188 | 0.138 | 0.347 | **0.000***** | 0.904 | 0.986 |

Supplementary Table S5. Pairwise differentiation estimates (F_ST_) among populations of white-bellied pangolins from Cameroon, Equatorial Guinea and Gabon (significant values in bold) based on 20 microsatellites loci.

Populations refer to Table S1. Significant values are in bold.

| **Populations** | **Bayomen** | **PNCM** | **Abong**  **Mbang** | **Eseka** | **Yabassi** | **Foumbot** | **GES** | **Manengole** | **Sangmelima** | **Mt Cam** | **Gabon** |
| --- | --- | --- | --- | --- | --- | --- | --- | --- | --- | --- | --- |
| **Bayomen** | 0.00000 |  |  |  |  |  |  |  |  |  |  |
| **PNCM** | **0.03976** | 0.00000 |  |  |  |  |  |  |  |  |  |
| **Abong Mbang** | 0.02465 | 0.00792 | 0.00000 |  |  |  |  |  |  |  |  |
| **Eseka** | 0.00525 | 0.01817 | 0.00593 | 0.00000 |  |  |  |  |  |  |  |
| **Yabassi** | 0.01378 | **0.04401** | **0.03349** | **0.02374** | 0.00000 |  |  |  |  |  |  |
| **Foumbot** | 0.00541 | **0.03251** | **0.02601** | **0.02152** | 0.01287 | 0.00000 |  |  |  |  |  |
| **GES** | **0.04852** | **0.03012** | **0.03201** | **0.03550** | **0.03378** | **0.03096** | 0.00000 |  |  |  |  |
| **Manengole** | 0.01116 | **0.02952** | **0.01930** | **0.01475** | 0.01659 | **0.02317** | **0.03240** | 0.00000 |  |  |  |
| **Sangmelima** | 0.02283 | 0.00608 | 0.00715 | 0.00862 | **0.02883** | **0.02175** | **0.02574** | **0.01550** | 0.00000 |  |  |
| **Mt Cam** | -0.01168 | 0.01783 | 0.01957 | 0.00610 | 0.00857 | 0.01539 | 0.01918 | -0.00132 | 0.01162 | 0.00000 |  |
| **Gabon** | **0.21310** | **0.20057** | **0.18398** | **0.19074** | **0.21153** | **0.22489** | **0.19854** | **0.20439** | **0.19015** | **0.18971** | 0.00000 |

Supplementary Table S6. Private alleles from seven loci potentially usable to trace the trade of white-bellied pangolins in western central Africa.

Number of individuals = 0 refers to private alleles characterizing a reference population but not found in any white-bellied pangolins from urban markets and seizures.

| **Locus** | **Population** | **Private allele (size)** | **Number of individuals assigned from urban markets and seizures** |
| --- | --- | --- | --- |
| PT1669238 | Mt Cameroon | **186** | 9 |
| PT2019332 | Abong Mbang | **186** | 2 |
| PT308752 | Manengole | **148** | 2 |
| PT308752 | Abong Mbang | **176** | 3 |
| PT308752 | South of Equatorial Guinea | **180** | 11 |
| PT308752 | Yabassi | **208** | 0 |
| PT338821 | Sangmelima | **256** | 5 |
| PT338821 | Abong Mbang | **268** | 1 |
| PT338821 | Sangmelima | **274** | 1 |
| PT338821 | Sangmelima | **282** | 1 |
| PT338821 | Sangmelima | **284** | 0 |
| PT34432 | Sangmelima | **109** | 0 |
| PT34432 | Eseka | **115** | 5 |
| PT34432 | Yabassi | **125** | 2 |
| PT34432 | Abong Mbang | **129** | 3 |
| PT34432 | Mt Cameroon | **184** | 0 |
| PT796077 | Mt Cameroon | **161** | 0 |
| PT839522 | Abong Mbang | **205** | 2 |

Supplementary Table S7. List of the 47 white-bellied pangolins from urban markets or international seizures traced to their reference populations in western central Africa.

| **Locus** | **Assigned individual** | **Market / Seizure** | **Source population** |  | **Locus** | **Assigned individual** | **Market / Seizure** | **Source population** |
| --- | --- | --- | --- | --- | --- | --- | --- | --- |
| **PT839522** | Dla A1 | Douala Central Market | Abong Mbang |  | **PT338821** | Y129 | Yaoundé Market | Sangmelima |
| **PT34432** | Dla B45 | Douala Dakat Market | Abong Mbang |  | **PT338821** | Y352 | Yaoundé Market | Sangmelima |
| **PT308752** | Dla B59 | Douala Dakat Market | Abong Mbang |  | **PT338821** | Y360 | Yaoundé Market | Sangmelima |
| **PT338821** | LL180926022B | Brussels Airport (Belgium) | Abong Mbang |  | **PT338821** | Y374 | Yaoundé Market | Sangmelima |
| **PT839522** | Y137 | Yaoundé Market | Abong Mbang |  | **PT338821** | Y77 | Yaoundé Market | Sangmelima |
| **PT34432** | Y146 | Yaoundé Market | Abong Mbang |  | **PT338821** | Y2 | Yaoundé Market | Sangmelima |
| **PT2019332** | Y216 | Yaoundé Market | Abong Mbang |  | **PT34432** | Dla B102 | Douala Dakat Market | Sangmelima |
| **PT308752** | Y354 | Yaoundé Market | Abong Mbang |  | **PT308752** | LL180926022A | Brussels Airport (Belgium) | South of Equatorial Guinea |
| **PT308752** | Y69 | Yaoundé Market | Abong Mbang |  | **PT308752** | Y162 | Yaoundé Market | South of Equatorial Guinea |
| **PT34432** | Y69 | Yaoundé Market | Abong Mbang |  | **PT308752** | Y221 | Yaoundé Market | South of Equatorial Guinea |
| **PT2019332** | Y90 | Yaoundé Market | Abong Mbang |  | **PT308752** | Y222 | Yaoundé Market | South of Equatorial Guinea |
| **PT308752** | Y100 | Yaoundé Market | Manengole |  | **PT308752** | Y228 | Yaoundé Market | South of Equatorial Guinea |
| **PT308752** | Y359 | Yaoundé Market | Manengole |  | **PT308752** | Y229 | Yaoundé Market | South of Equatorial Guinea |
| **PT1669238** | Dla B13 | Douala Dakat Market | Mt Cameroon |  | **PT308752** | Y336 | Yaoundé Market | South of Equatorial Guinea |
| **PT1669238** | Dla B85 | Douala Dakat Market | Mt Cameroon |  | **PT308752** | Y342 | Yaoundé Market | South of Equatorial Guinea |
| **PT1669238** | Y109 | Yaoundé Market | Mt Cameroon |  | **PT308752** | Dla B100 | Douala Dakat Market | South of Equatorial Guinea |
| **PT1669238** | Y179 | Yaoundé Market | Mt Cameroon |  | **PT308752** | Dla B101 | Douala Dakat Market | South of Equatorial Guinea |
| **PT1669238** | Y251 | Yaoundé Market | Mt Cameroon |  | **PT308752** | Y332 | Yaoundé Market | South of Equatorial Guinea |
| **PT1669238** | Y33 | Yaoundé Market | Mt Cameroon |  | **PT34432** | Y157 | Yaoundé Market | Eseka |
| **PT1669238** | Y349 | Yaoundé Market | Mt Cameroon |  | **PT34432** | Y196 | Yaoundé Market | Eseka |
| **PT1669238** | Y361 | Yaoundé Market | Mt Cameroon |  | **PT34432** | Y232 | Yaoundé Market | Eseka |
| **PT1669238** | Y64 | Yaoundé Market | Mt Cameroon |  | **PT34432** | Y242 | Yaoundé Market | Eseka |
| **PT34432** | Dla A30 | Douala Central Market | Yabassi |  | **PT34432** | Y96 | Yaoundé Market | Eseka |
| **PT34432** | Dla B3 | Douala Dakat Market | Yabassi |  |  | | | |

Supplementary Figure S1. Map of the 40 bushmeat markets and forest sites surveyed in Cameroon, Equatorial Guinea and Gabon. The map was drawn using ArcMap of ArcGIS for Desktop 10.1 (<http://www.esri.com/software/arcgis/arcgis-for-desktop>).

S1-Abong Mbang; S2-Akonolinga; S3-Bangong; S4-Bayib Assibong; S5-Bioko; S6-Bipindi; S7-Campo; S8-Douala Central Market; S9-Douala Dakat Market; S10-Djoum; S11-Ekombitié; S12-Eseka; S13-Esse; S14-Foumbot; S15-Bitam; S16-Anguma; S17-Bongoro; S18-Ebenguan; S19-Emangos; S20-Misergue; S21-Taguete; S22-Makokou; S23-Medouneu; S24-Lolodorf; S25-Maan; S26-Mamb; S27-Manengole; S28-Manyemen; S29-Nditam; S30-Sangmelima; S31-Seizure; S32-Yabassi; S33-Yaoundé Nkolndongo Market; S34-Yokadouma; S35-Franceville; S36-Mocabe; S37-Nkoltang; S38-Okoumbi; S39-Oyem; S40-Tchibanga.


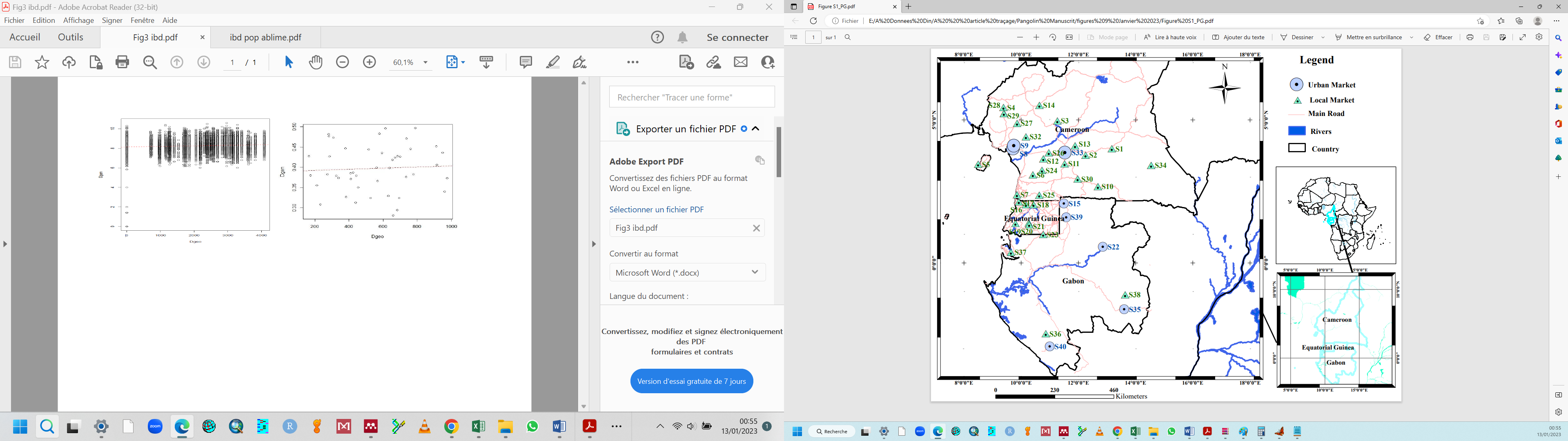


Supplementary Figure S2. Neighbor joining tree of white-bellied pangolins based on 641 cytochrome *b* sequences. [pdf file]

Bootstrap values > 75% are shown at nodes. Sequence clusters are taxonomically delimitated according to Gaubert et al. (2016). Sample numbering corresponds to Table S1. Scale bar at bottom represents percentage of K2P distance. **** designates the outgroup.

Supplementary Figure S4. Median-joining network showing mutational relationships between the 74 haplotypes observed in 553 white-bellied pangolins from Cameroon (yellow), Equatorial Guinea (continent; blue), Bioko Island (green), Roissy airport – France (grey), Brussels airport – Belgium (purple), and Gabon (pink).

Size of circle is proportional to the number of haplotypes (e.g., H_30 = 1). Mutational steps are illustrated as dashes perpendicular to network connections.


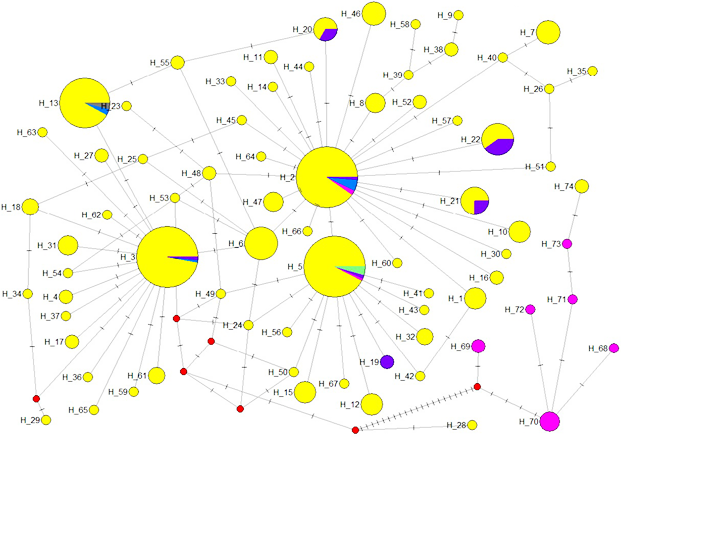


Supplementary Figure S5. Distribution of cytochrome b haplotypes among white-bellied pangolins from the Western Central Africa and Gabon lineages. The map was drawn using ArcMap of ArcGIS for Desktop 10.1 (<http://www.esri.com/software/arcgis/arcgis-for-desktop>).

Site numbers refer to Figure S1. H1-H67 = Western Central Africa; H68-H74 = Gabon.


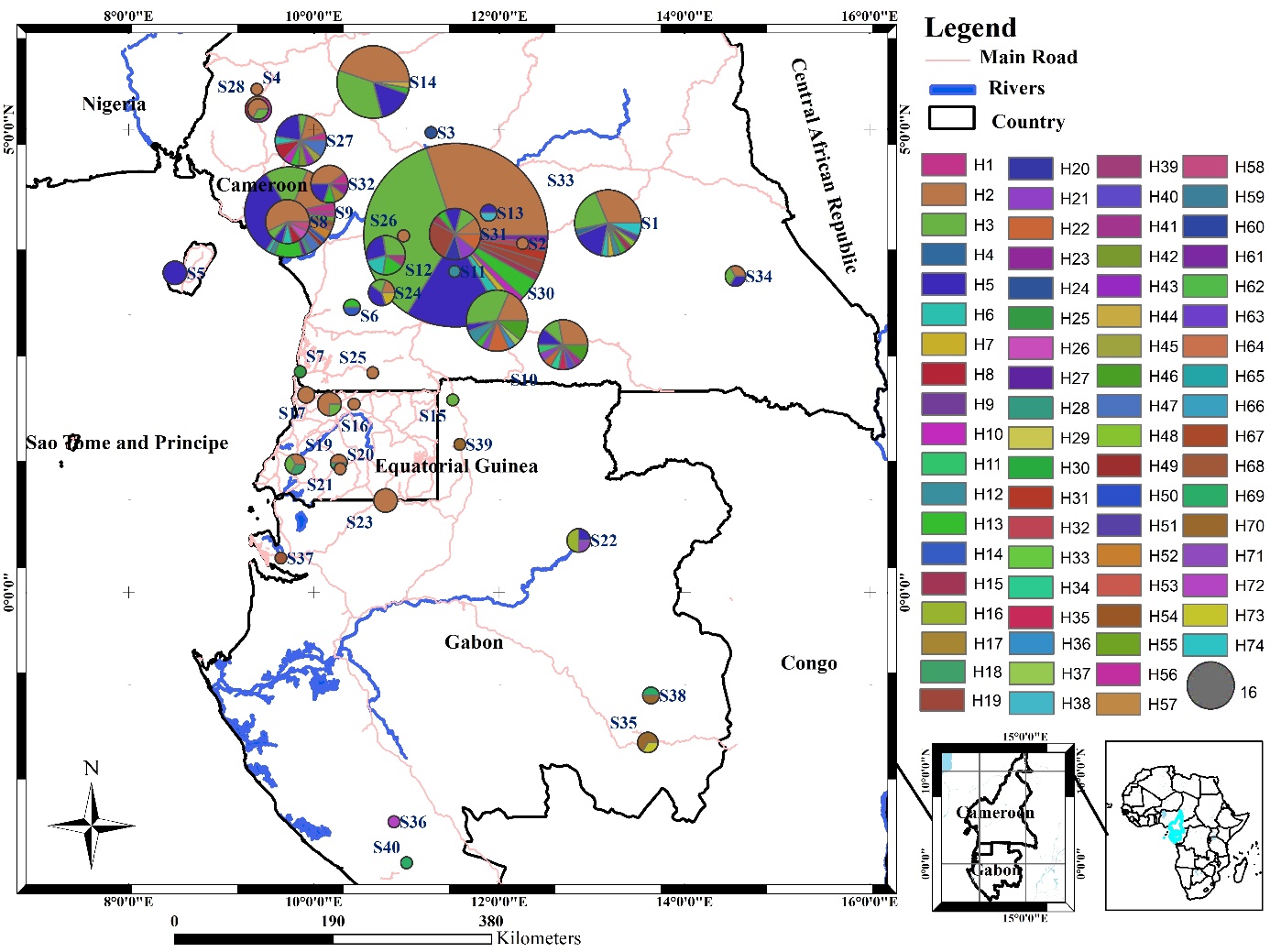


Supplementary Figure S6. Plot of mismatch distribution for white-bellied pangolins from WCA under hypothesis of sudden demographic expansion (left) and spatial demographic expansion (right).

Frequencies expected in red; Frequencies observed in green.


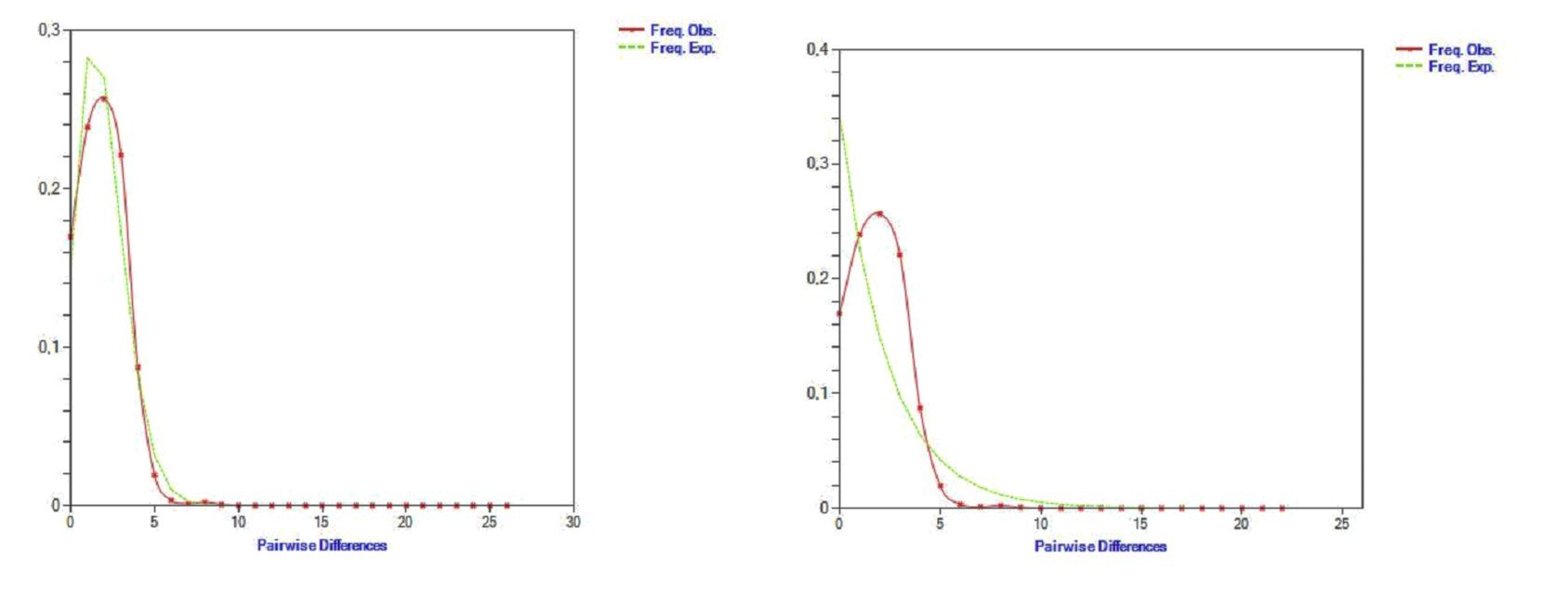


Supplementary Figure S7. Distribution of genetic variance (PCoA) among populations of white-bellied pangolins from WCA and Gab lineages.

(**a**) = including urban markets; (**b**) = excluding urban markets. Percentage of explained variance per axis is given between brackets.

Population names refer to Table S1. DM = Douala Markets; YNM = Yaoundé Market.

(**a**)

(**b**)

Supplementary Figure S8. Assignment plots among white-bellied pangolins from western central Africa (N = 558) as assessed with STRUCTURE for K = 2 to K = 10. All the samples from urban markets, seizures and reference populations were included.

Each individual is represented by a vertical bar. The colour of STRUCTURE plot represents nuclear clusters concordant with mitochondrial lineage assignment (blue = WCA; orange = Gab). 1- Individuals mtDNA-assigned to WCA lineage; 2- Individuals mtDNA-assigned to Gab lineage.


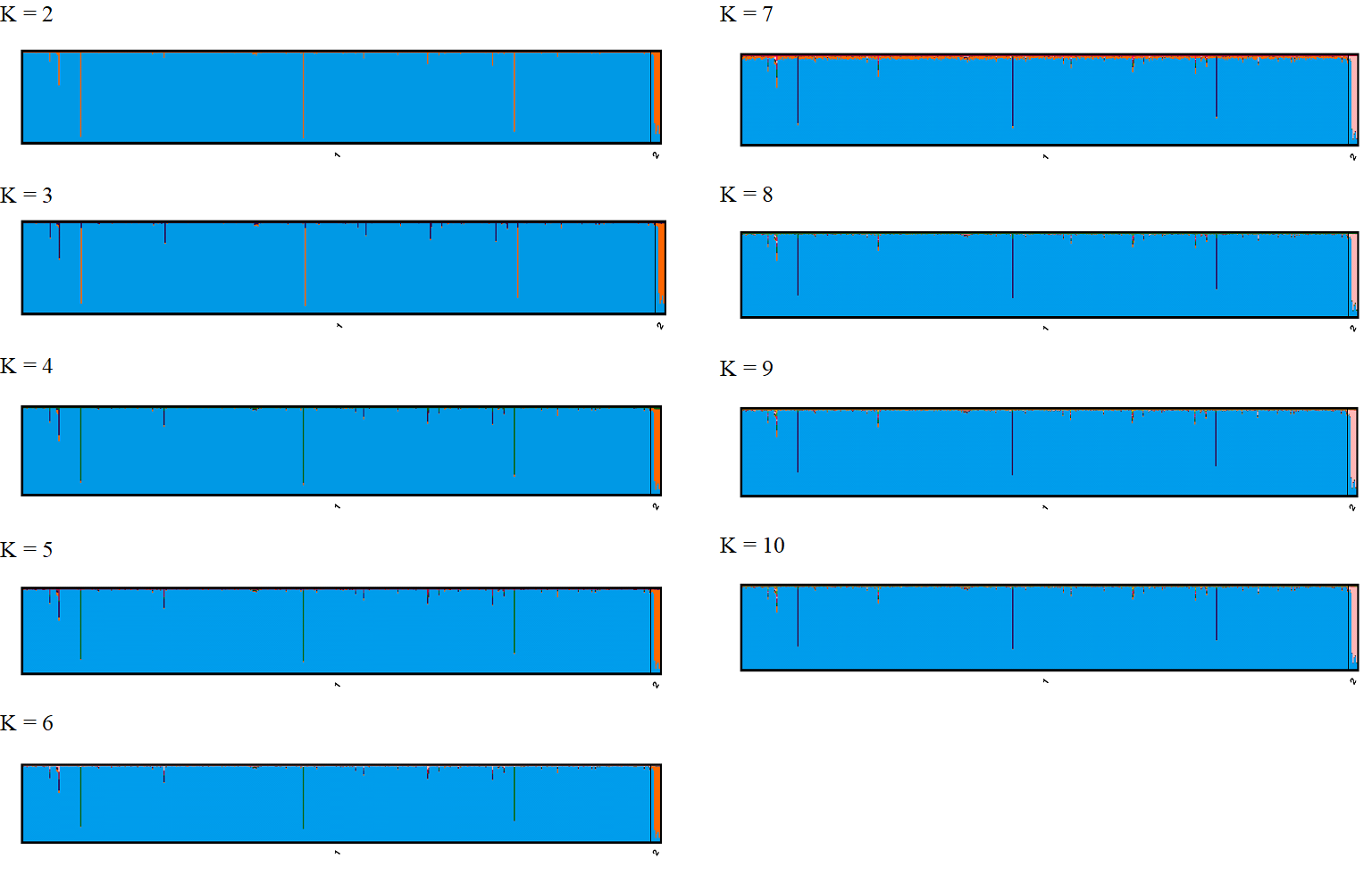


Supplementary Figure S9. Assignment plots among white-bellied pangolins from the WCA lineage (N = 181) as assessed with STRUCTURE for K = 2 to K = 10. Only samples from 10 reference populations were included.

Each individual is represented by a vertical bar. Reference populations: 1-Bayomen; 2-PNCM; 3-Abong Mbang; 4-Eseka; 5-Yabassi; 6-Foumbot; 7-GES; 8-Manengole; 9-Sangmelima; 10-Mt Cam.


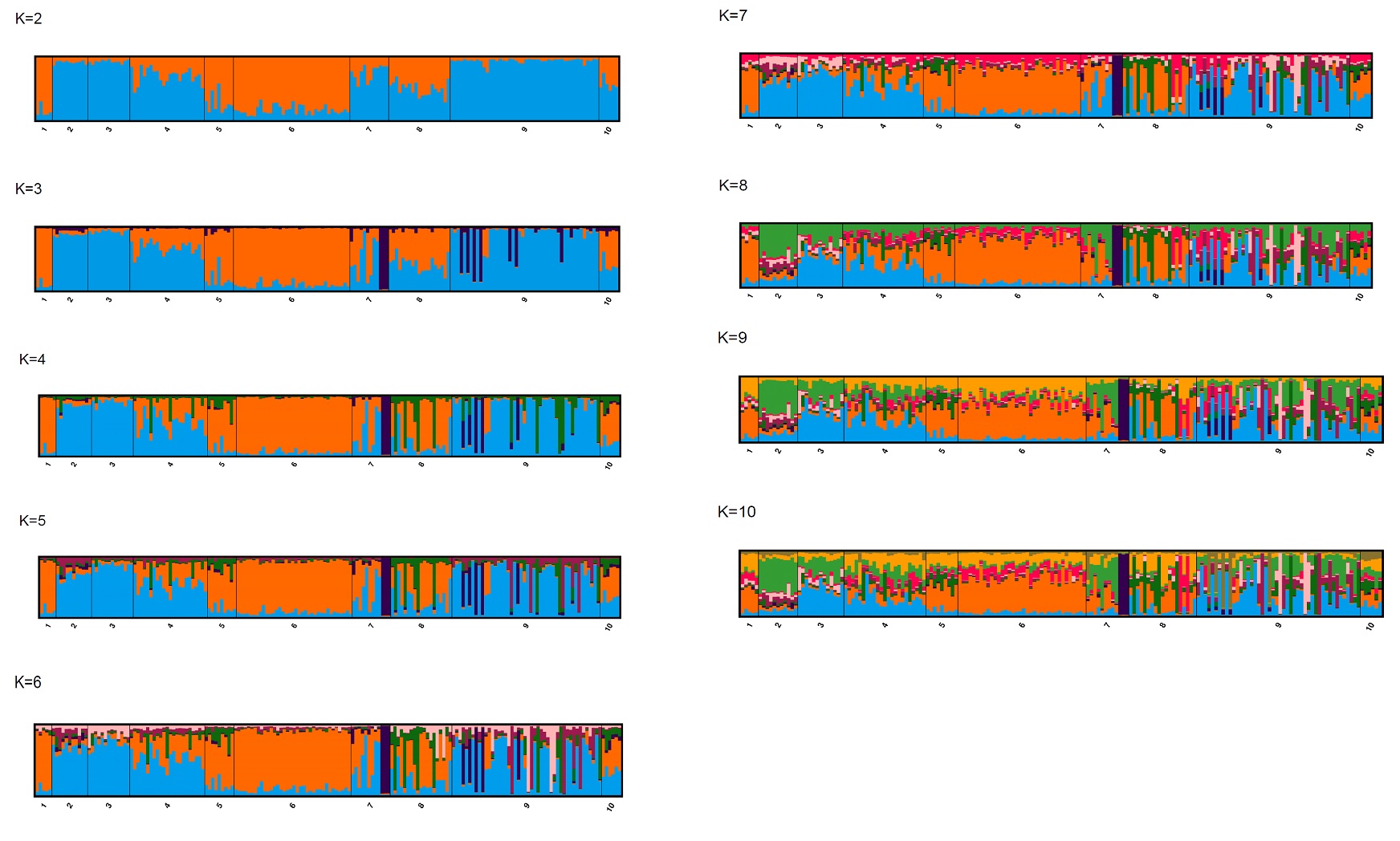


Supplementary Figure S10. Spatial clustering of white-belled pangolins in the WCA lineage obtained using Geneland for K = 1 to K = 25 (left) and K = 10 to K = 13 (right).

A-Map of population membership at K = 11; Coloured areas refer to the geographic or spatial spread of the individuals; Black dots represent individuals. B-Estimated number of populations obtained.


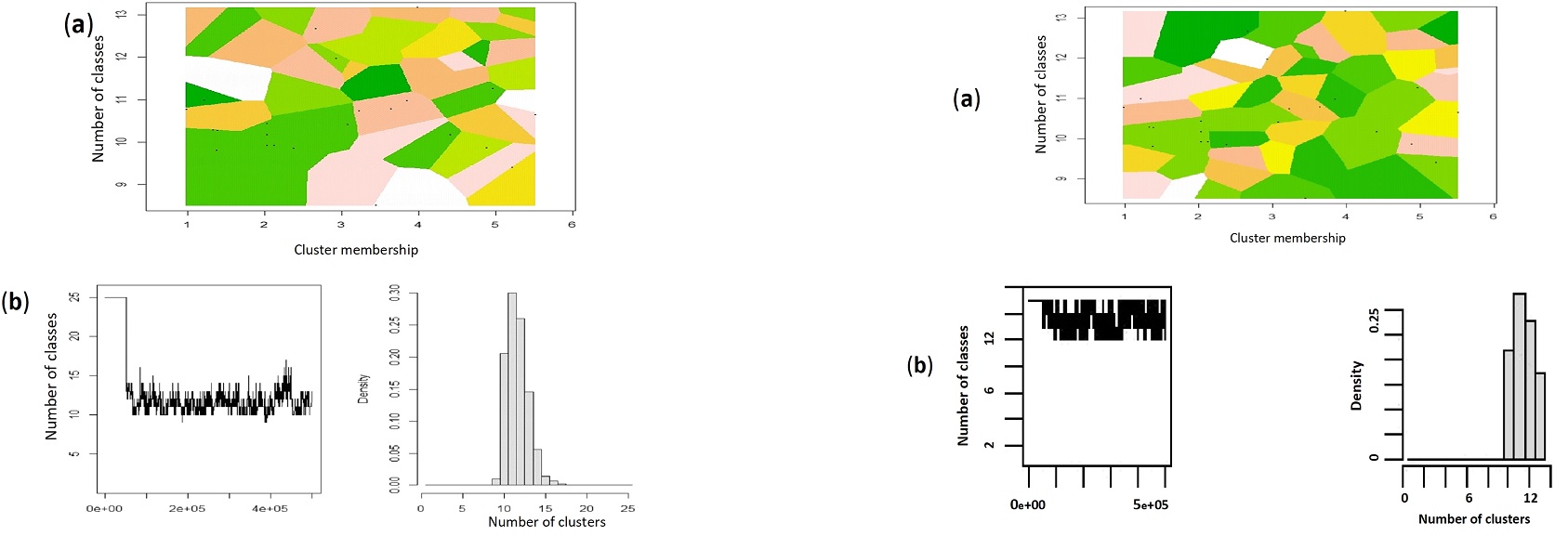


Supplementary Figure S11. Isolation by distance among (**a**) individuals and (**b**) populations of white-bellied pangolins from continental Western Central Africa as inferred from 20 microsatellites loci. Dashed curve indicates linear regression. Dgen-Genetic distance; Dgeo-Geographic distance.


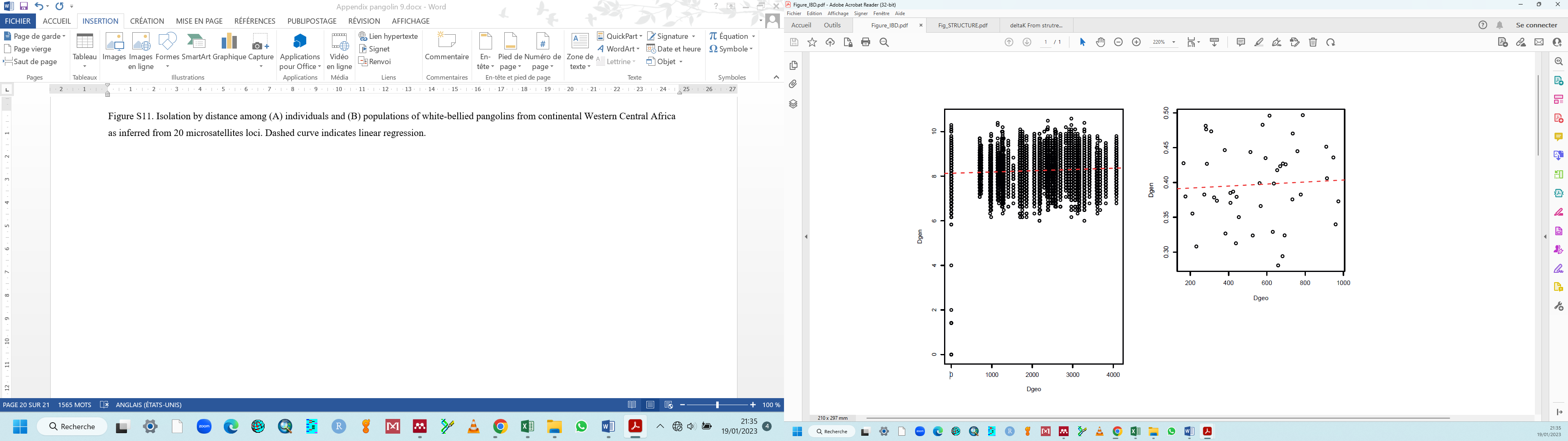


**b**

**a**

Supplementary Figure S12. Unbiased probability of identity (uPI) and probability of identity among siblings (PIsibs) for increasing, optimized combinations among the 20 microsatellite markers.
