# Supplementary figures and images for: Genetic tracing of the illegal trade of the white-bellied pangolin (Phataginus tricuspis) in western Central Africa
Source: Sci Rep. 2024 Jun 7;14:13131. doi: 10.1038/s41598-024-63666-9 (PMC11161582; doi:10.1038/s41598-024-63666-9)

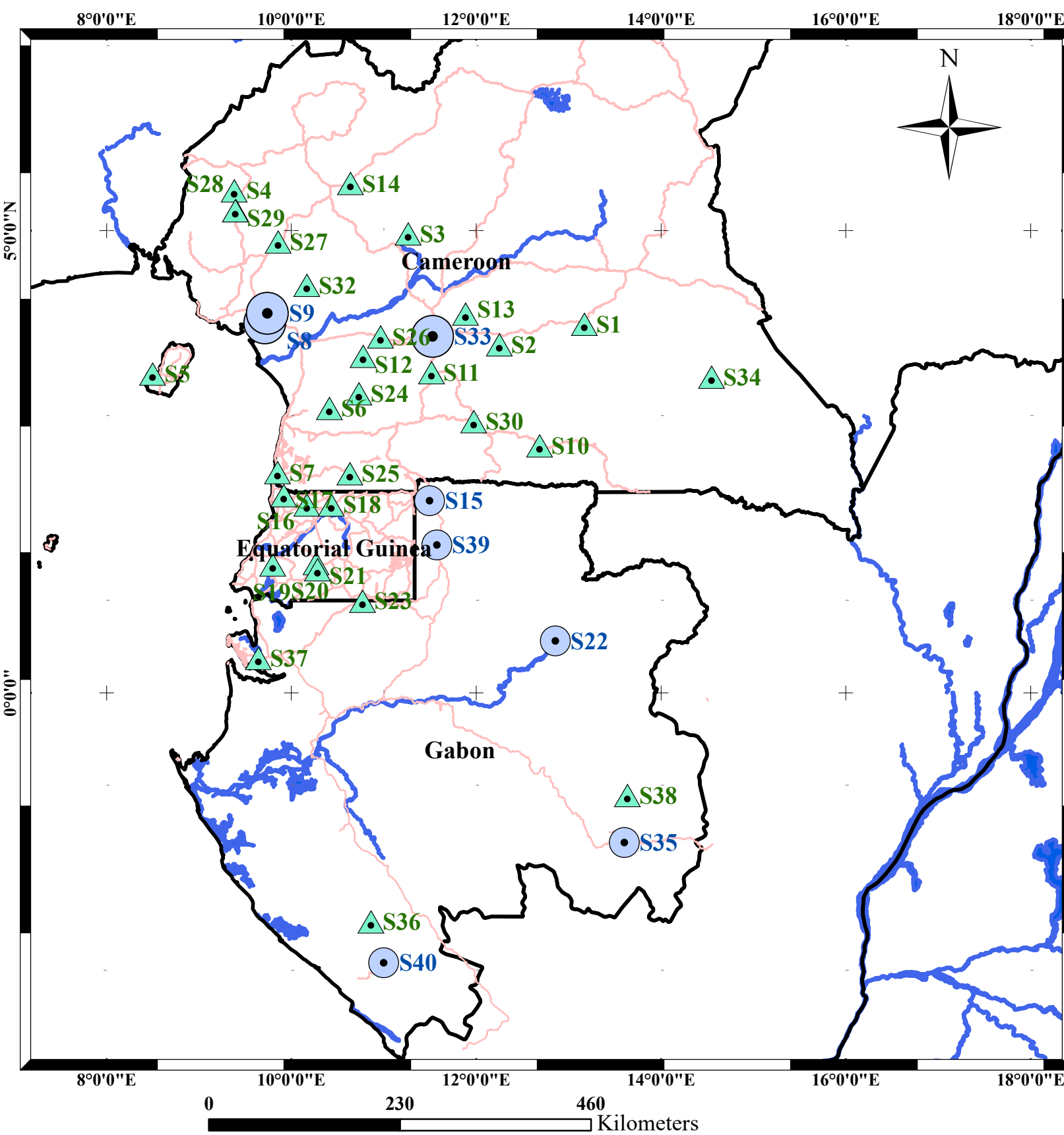

## Legend

- Urban Market
- Local Market
- Main Road
- Rivers
- Country

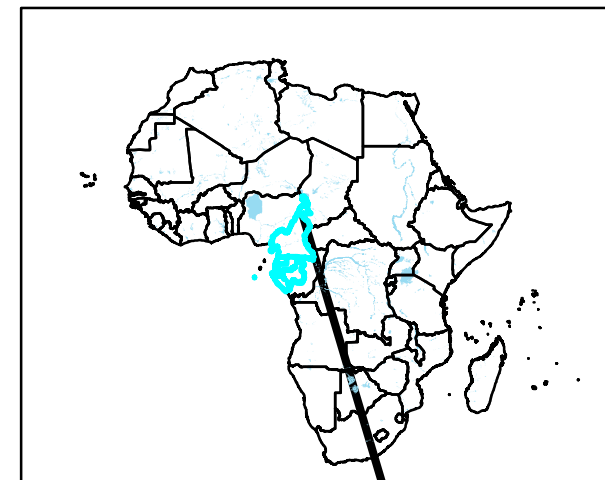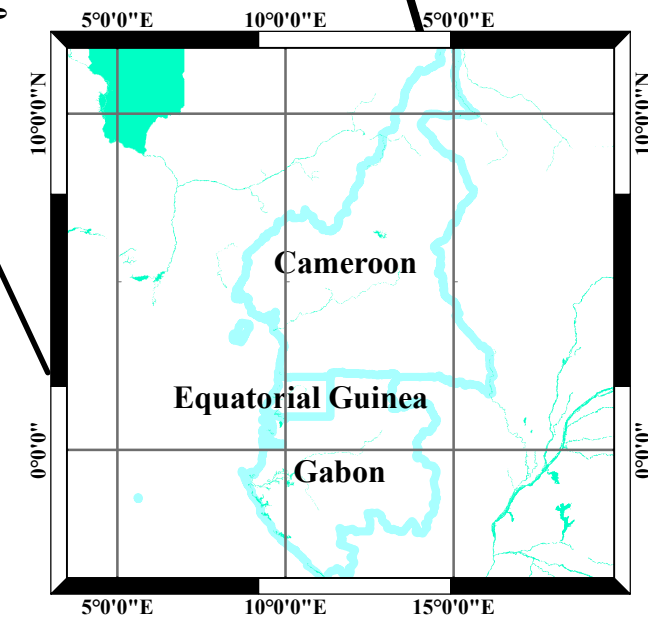

Supplement: Supplementary file 2 — Supplementary Information 2. [file 41598_2024_63666_MOESM2_ESM.pdf]
