# Supplementary material for: Genetic tracing of the illegal trade of the white-bellied pangolin (Phataginus tricuspis) in western Central Africa
Source: Sci Rep. 2024 Jun 7;14:13131. doi: 10.1038/s41598-024-63666-9 (PMC11161582; doi:10.1038/s41598-024-63666-9)
Supplement: Supplementary file 3 — Supplementary Information 3. [file 41598_2024_63666_MOESM3_ESM.pdf]

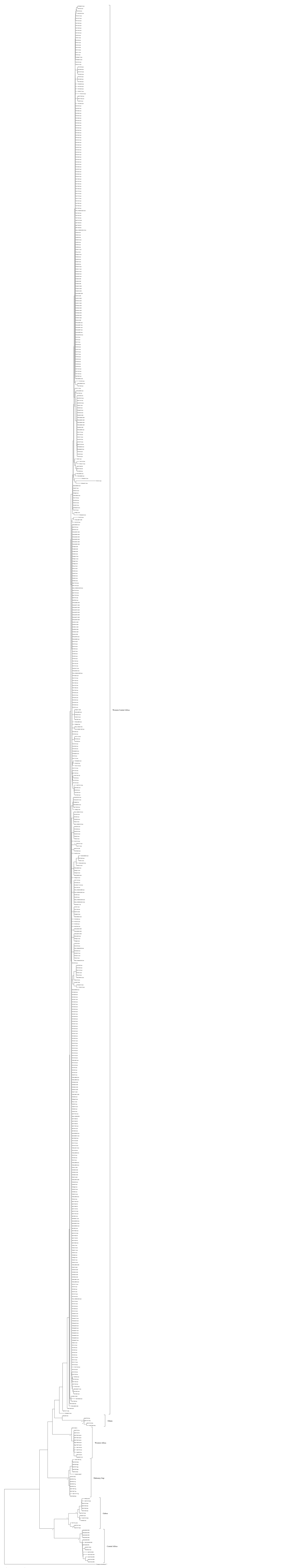

Western Central Africa
Ghana
Western Africa
Dahomey Gap
Gabon
Central Africa
